# Acute respiratory distress syndrome (ARDS)-associated acute cor pulmonale and patent foramen ovale: a multicenter noninvasive hemodynamic study

**DOI:** 10.1186/s13054-015-0898-5

**Published:** 2015-04-17

**Authors:** Annick Legras, Agnès Caille, Emmanuelle Begot, Gwenaëlle Lhéritier, Thierry Lherm, Armelle Mathonnet, Jean-Pierre Frat, Anne Courte, Laurent Martin-Lefèvre, Jean-Paul Gouëllo, Emmanuelle Mercier, Philippe Vignon, on behalf of the ARCO and CRICS network

**Affiliations:** Medical ICU, Teaching hospital of Tours, 2 Bd Tonnellé, 37044 Tours, cedex 9 France; Inserm, CIC 1415, CHRU de Tours, 2, boulevard Tonnellé, 37044 Tours, cedex 9 France; Université François-Rabelais. PRES Centre-Val de Loire, 60 rue du Plat d’Etain, 37020 Tours, cedex 1 France; Medical-Surgical ICU, Teaching hospital of Limoges, 2 Av Martin Luther King, 87042 Limoges cedex 1, France; INSERM, CIC1435, 2 Av Martin Luther King, 87042 Limoges, cedex 1 France; Medical ICU, Hospital of Chartres, 4 rue Claude Bernard, 28630 Coudray, France; Medical ICU, Hospital of Orléans, 14 Av de l’Hôpital, 45100 Orléans, France; Medical ICU, Teaching hospital of Poitiers, 2 route de la Milétrie CS 90577, 86021 Poitiers, cedex France; Medical-surgical ICU, Hospital of Saint-Brieuc, 10 rue Marcel Proust, 22000 Saint-Brieuc, France; Medical ICU, Hospital of La Roche-sur-Yon, Les Oudairies, 85925 La Roche-sur-Yon, cedex 9 France; Medical-surgical ICU, Hospital of Saint-Malo, 1 rue Marne, 35400 Saint-Malo, France; University of Limoges, 39E Rue Camille Guérin, 87000 Limoges, France

## Abstract

**Introduction:**

Acute cor pulmonale (ACP) and patent foramen ovale (PFO) remain common in patients under protective ventilation for acute respiratory distress syndrome (ARDS). We sought to describe the hemodynamic profile associated with either ACP or PFO, or both, during the early course of moderate-to-severe ARDS using echocardiography.

**Methods:**

In this 32-month prospective multicenter study, 195 patients with moderate-to-severe ARDS were assessed using echocardiography during the first 48 h of admission (age: 56 (SD: 15) years; Simplified Acute Physiology Score: 46 (17); PaO_2_/FiO_2_: 115 (39); V_T_: 6.5 (1.7) mL/kg; PEEP: 11 (3) cmH_2_O; driving pressure: 15 (5) cmH_2_O). ACP was defined by the association of right ventricular (RV) dilatation and systolic paradoxical ventricular septal motion. PFO was detected during a contrast study using agitated saline in the transesophageal bicaval view.

**Results:**

ACP was present in 36 patients, PFO in 21 patients, both PFO and ACP in 8 patients and the 130 remaining patients had neither PFO nor ACP. Patients with ACP exhibited a restricted left ventricle (LV) secondary to RV dilatation and had concomitant RV dysfunction, irrespective of associated PFO, but preserved LV systolic function. Despite elevated systolic pulmonary artery pressure (sPAP), patients with isolated PFO had a normal RV systolic function. sPAP and PaCO_2_ levels were significantly correlated.

**Conclusions:**

In patients under protective mechanical ventilation with moderate-to-severe ARDS, ACP was associated with LV restriction and RV failure, whether PFO was present or not. Despite elevated sPAP, PFO shunting was associated with preserved RV systolic function.

## Introduction

In patients with the acute respiratory distress syndrome (ARDS), acute right ventricular (RV) afterloading secondary to increased pulmonary vascular resistance may result in acute cor pulmonale (ACP) or patent foramen ovale (PFO). Despite the use of protective mechanical ventilation, the prevalence of ACP and PFO has recently been reported to range between 22 and 25%, and 16 and 19% of ARDS patients, respectively [1-4]. PFO shunting may worsen ARDS-induced hypoxemia, thereby limiting the beneficial effects of recruitment maneuvers such as positive end-expiratory pressure (PEEP) trials [2]. In contrast, PFO may reduce the deleterious effects of elevated pulmonary vascular resistance on RV systolic function. Indeed, balloon atrial septostomy is a proposed procedure aimed at decompressing right cardiac pressures and increasing left ventricular (LV) preload and cardiac output in patients with severe pulmonary hypertension and associated afterloaded RV [5].

Transesophageal echocardiography (TEE) is more accurate than transthoracic echocardiography (TTE) for the diagnosis of ACP and PFO in mechanically ventilated patients with ARDS [3]. Noninvasive hemodynamic assessment using echocardiography in ventilated patients with ARDS and associated ACP or PFO have yet been scarcely reported, and solely in single-center studies [1,2,6]. Specifically, the potential influence of PFO on RV function in ARDS patients, with respect to its association with ACP or not, has not yet been elucidated. Accordingly, we sought to describe the hemodynamic profile in patients who develop ACP and shunting through a PFO in a large, multicenter, previously described population of mechanically ventilated patients during the early course of moderate-to-severe ARDS [3].

## Material and methods

### Patients

This prospective observational study was conducted in nine intensive care units (ICUs) and was approved by the ethics committee of the *Société de Réanimation de Langue Française*, which waived informed consent since it was in accordance with the standard of care of participating centers. Between November 2009 and June 2012, all patients with ARDS, as defined by the American-European consensus conference [7] modified for one criterion (partial pressure of arterial oxygen/fraction of inspired oxygen (PaO_2_/FiO_2_) ≤200 with a FiO_2_ of 1 and PEEP ≥5 cm H_2_O) were screened to participate in the study. Cardiogenic pulmonary edema was ruled out by the depiction of low LV filling pressure during echocardiographic assessment, as reflected by a lateral E/E’ ratio <8 [8]. Since this study was performed prior to the recent Berlin definition of ARDS [9], we secondarily classified the severity of ARDS-induced hypoxemia in our patients accordingly.

### Echocardiography

TTE and TEE examinations were all performed by intensivists highly trained in critical care echocardiography [10]. In the long-axis view of the heart (TEE four-chamber view), RV end-diastolic area (RVEDA), RV end-systolic area (RVESA), LV end-diastolic area (LVEDA), and LV ejection fraction (LVEF) using the modified Simpson’s rule were measured. RV fractional area change (RVFAC) was computed as RVEDA - RVESA/RVEDA and expressed as a percentage. In the short-axis view of the heart (transgastric short-axis view at the level of the papillary muscles), the ventricular septal motion was analyzed throughout the cardiac cycle. The LV eccentricity index was measured both at end-systole and at end-diastole [11]. The 100 to 120° transgastric view allowed the measurement of LV outflow tract (LVOT) Doppler flow velocity time integral. A pulse wave Doppler sample was located to solely obtain the closing click of the aortic valve, with the best alignment with the systolic LV outflow [12]. The LVOT diameter was measured at the level of the aortic cusps insertion in the transesophageal 120° view zoomed on the initial ascending aorta, and the orifice area was calculated as Π x (LVOT diameter)^2^/4. The LV stroke volume (LVSV) was calculated as LVOT area x LVOT Doppler flow velocity time integral [12], and the cardiac index was computed. The maximal velocity of tricuspid regurgitation (Vmax TR) and M-mode tricuspid annular plane systolic elevation (TAPSE) were measured in the apical four-chamber view [13]. Systolic pulmonary artery pressure (sPAP) was obtained using the simplified Bernouilli’s equation: 4 x (Vmax TR)^2^ + central venous pressure (CVP). To reduce the lack of precision of CVP estimation based on the size of the inferior vena cava [14], CVP was invasively measured through central venous catheters. For each parameter, three non-consecutive measurements were performed at end-expiration and averaged. In previous studies, the interobserver variability in the measurement of pulse wave Doppler indices ranged between 1 and 13% [15], and the interobserver variability in the measurement of two-dimensional parameters was less than 10% [16].

ACP was defined by the association of RV dilatation in the long-axis view of the heart (RVEDA/LVEDA >0.6) and a visually identified systolic paradoxical ventricular septal motion in the short-axis view of the heart [17]. PFO was detected during a contrast study using agitated saline in the TEE bicaval view, without provoking maneuvers such as end-inspiratory breath holding or sudden release of PEEP [18]. PFO shunting was defined as the issue of microcavitations from the right to the left atrium within three cardiac cycles after the full opacification of the right atrium [3].

### Statistical analysis

Patients were divided into the four following groups: presence of both a PFO and ACP, presence of isolated PFO, presence of isolated ACP, and absence of both PFO and ACP. Categorical variables were reported as numbers and percentages. Continuous variables were expressed as mean and standard deviation (SD) or median and 25^th^ to 75^th^ percentiles. Comparisons between the four groups were performed with Fisher’s exact test for binary variables and Kruskal-Wallis test for continuous variables. The correlation between individual values of partial pressure of arterial carbon dioxide (PaCO_2_) and sPAP was calculated using a Pearson correlation coefficient. A *P* value <0.05 was considered statistically significant.

## Results

Of 201 patients with ARDS included in the study, the echocardiographic examination was not available for off-line measurements in 6 patients. Finally, 195 patients were studied (age: 56 (SD: 15) years; Simplified Acute Physiology Score: 46 (17); PaO_2_/FiO_2_: 115 (39); tidal volume (V_T_): 6.5 (1.7) mL/kg; PEEP: 11 (3) cmH_2_O; driving pressure: 15 (5) cmH_2_O). ARDS was mainly related to infective pneumonia (62%) and was associated with a global 28-day mortality rate of 23% (95% confidence interval (CI): 17% to 30%). ACP was present in 36 patients, PFO in 21 patients, both PFO and ACP in 8 patients and the 130 remaining patients had neither PFO nor ACP.

**Figure 1:**
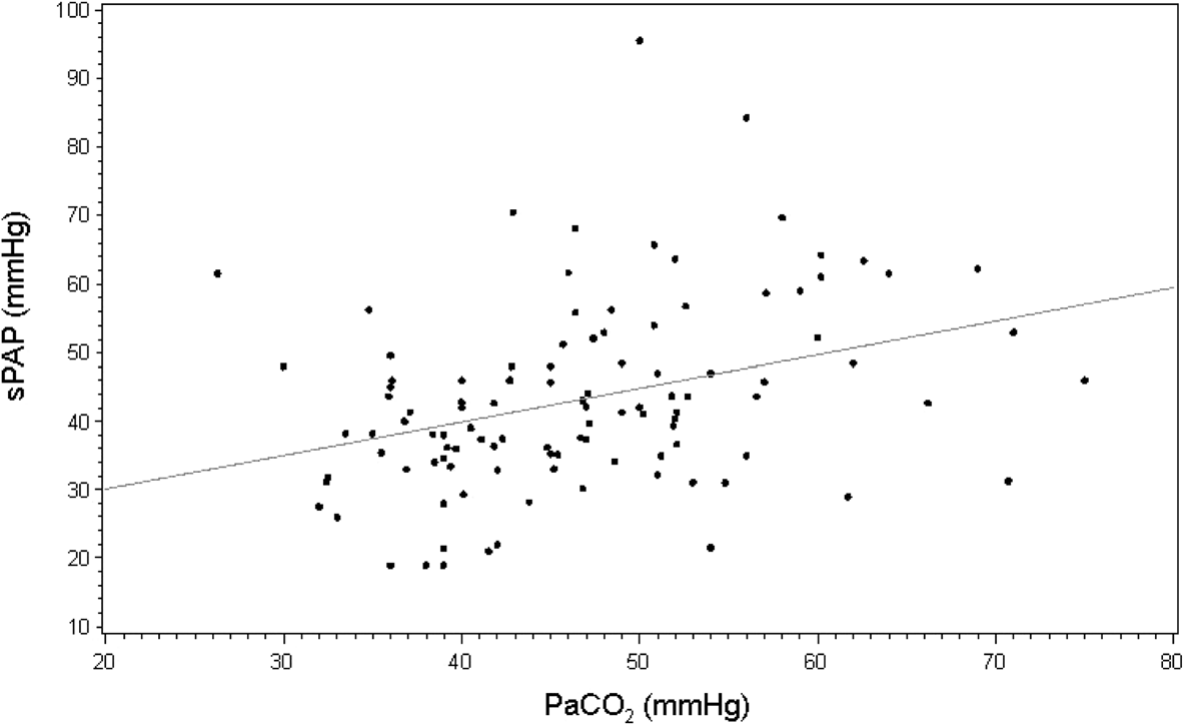
Relationship between individual values of PaCO_2_ and systolic pulmonary artery pressure (sPAP) measured in moderate-to-severe ARDS patients under protective ventilation using continuous wave Doppler interrogation of tricuspid regurgitant jet, when present. ARDS, acute respiratory distress syndrome; PaCO_2_, partial pressure of arterial carbon dioxide.

Respiratory and circulatory parameters were not statistically different between groups in these patients with moderate-to-severe ARDS who underwent protective ventilation and frequently received vasopressor support. Prone positioning was used more frequently in patients with isolated ACP than in other groups (Table 1). When compared to patients with isolated PFO or ACP, patients with both PFO and ACP exhibited greater RV dilatation, as reflected by a higher median RVEDA/LVEDA ratio (Table 2). LV end-systolic eccentricity index was significantly higher in ACP patients, irrespective of associated PFO. In contrast, LV end-diastolic eccentricity index did not differ between groups. Patients with ACP exhibited LV restriction, as reflected by a significantly lower median LV end-diastolic volume (LVEDV), and tended to have lower LVSV, whether PFO was associated or not (Table 2). LVEF was uniformly preserved across groups. In contrast, RV systolic function was reduced in ACP patients, irrespective of associated PFO, as reflected by significantly lower median values of RVFAC and TAPSE. Despite substantially elevated sPAP, patients with isolated PFO had preserved RV function, with similar median values of RVFAC and TAPSE than those of patients without PFO and ACP (Table 2). Median sPAP was significantly higher in patients with a PaCO_2_ > 60 mmHg (51 ± 13 vs. 42 ± 13 mmHg: *P* = 0.04). A significant correlation was found between sPAP and PaCO_2_ levels in the study population (r: 0.35; *P* = 0.0002) (Figure 1).

**Table 1:** **Respiratory and hemodynamic variables according to the presence or absence of ACP and/or PFO** ^**a**^

**Parameters**	**PFO and ACP**	**PFO only**	**ACP only**	**No PFO and no ACP**	***P*** **value**
	**(n = 8)**	**(n = 21)**	**(n = 36)**	**(n = 130)**	
V_T_/predicted body weight (mL/kg)	6.5 [5; 7]	6 [5; 7]	6.5 [6; 8]	6 [5; 7]	0.64
PEEP (cmH_2_O)	10 [6; 13]	10 [8; 12]	10 [8; 14]	11 [8; 12]	0.36
Driving pressure (cmH_2_O)^b^	15 [13; 16]	16 [13; 17]	15 [12; 20]	14 [11; 17]	0.22
PaO_2_/FiO_2_	95 [75; 115]	107 [71; 153]	114 [72; 145]	112 [91; 154]	0.33
PaCO_2_ (mmHg)	47 [40; 62]	48 [41; 52]	50 [44; 58]	45 [39; 53]	0.28
Heart rate (bpm)	106 [91; 116]	105 [97; 128]	97 [85; 113]	100 [85; 116]	0.29
mBP (mmHg)	79 [70; 85]	79 [70; 89]	80 [74; 88]	81 [73; 90]	0.80
CVP (mmHg)	11 [10; 13]	11 [10; 12]	11 [10; 14]	10 [9; 12]	0.96
Lactates (mmol/L)	1.3 [1.2; 1.9]	1.3 [1; 2]	1.3 [1; 2.1]	1.4 [1.1; 2]	0.93
Prone positioning (n)	2 (25%)	3 (14%)	18 (50%)	44 (34%)	0.04
Nitric oxide (n)	3 (38%)	2 (10%)	6 (17%)	14 (11%)	0.15
Vasopressor support (n)	6 (75%)	10 (48%)	18 (50%)	62 (48%)	0.55
28-day mortality (n)	1 (13%)	6 (29%)	9 (25%)	29 (22%)	0.82

^a^Results are expressed as median values with 25^th^ to 75^th^ percentiles unless otherwise stated; ^b^defined as the difference between the inspiratory plateau pressure and positive end-expiratory pressure. ACP, acute cor pulmonale; PFO, patent foramen ovale; VT, tidal volume; PEEP, positive end-expiratory pressure; PaO2, partial pressure of arterial oxygen; FiO2, fraction of inspired oxygen; PaCO2, partial pressure of arterial carbon dioxide; mBP, mean blood pressure; CVP, central venous pressure.

**Table 2:** **Echocardiographic findings according to the presence or absence of ACP and/or patent foramen ovale PFO** ^**a**^

**Parameters**	**PFO and ACP**	**PFO only**	**ACP only**	**No PFO and no ACP**	***P*** **value**
	**(n = 8)**	**(n = 21)**	**(n = 36)**	**(n = 130)**	
RVEDA/LVEDA (cm^2^)	0.88 [0.69; 1.34]	0.71 [0.57; 0.77]	0.75 [0.64; 0.92]	0.62 [0.52; 0.72]	0.0002
LV eccentricity index at end-systole	1.32 [1.3; 1.44]	1.07 [1.02; 1.09]	1.33 [1.27; 1.51]	1.06 [1; 1.11]	<0.0001
LV eccentricity index at end-diastole	1.13 [1.07; 1.2]	1.08 [1.01; 1.14]	1.09 [1.04; 1.18]	1.07 [1.01; 1.13]	0.21
Cardiac index (L/min/m^2^)	3.58 [2.78; 3.71]	3.28 [2.84; 4.18]	2.93 [2.62; 3.37]	3.16 [2.6; 3.98]	0.27
LV stroke volume (mL)	59 [46; 68]	61 [48; 76]	58 [49; 61]	63 [52; 73]	0.09
LVEDV (mL)	67 [51; 85]	85 [54; 100]	79 [66; 93]	87 [68; 106]	0.04
LVEF (%)	65 [55; 74]	62 [46; 70]	58 [48; 66]	56 [44; 68]	0.60
RVFAC (%)	18 [15; 25]	32 [24; 40]	26 [17; 34]	34 [26; 42]	0.0007
TAPSE (mm)	15 [14; 20]	20 [17; 23]	18 [16; 20]	20 [16; 23]	0.02
Doppler-derived sPAP (mmHg)	46 [41; 60]	50 [39; 67]	45 [37; 55]	42 [35; 49]	0.17

^a^Results are expressed as median values with 25^th^ to 75^th^ percentiles. ACP, acute cor pulmonale; PFO, patent foramen ovale; RVEDA, right ventricular end-diastolic area; LVEDA, left ventricular end-diastolic area; LV, left ventricle; LVEDV, left ventricular end-diastolic volume; LVEF, left ventricular ejection fraction; RVFAC, right ventricular fractional area change; TAPSE, tricuspid annular plane systolic elevation; sPAP, systolic pulmonary artery pressure.

## Discussion

In this large population of patients ventilated for moderate-to-severe ARDS, ACP was associated with LV restriction and depressed RV systolic function, whether it was associated with PFO or not. ACP is the most severe presentation of RV failure secondary to an abrupt rise in RV afterload [17], such as that resulting from increased pulmonary vascular resistance associated with ARDS [19]. In our ACP patients, RV dilatation resulted in LV restriction within the stiff pericardial sac [17], as reflected by the significantly reduced median LVEDV, irrespective of associated PFO. This decrease of LV preload due to ventricular interaction presumably accounted for the observed trend of lower LVSV measured in ACP patients, since those patients had otherwise preserved LV systolic function, as reflected by normal LVEF. Since LVEF is internally normalized by preload (that is, LVEDV), median values were similar and uniformly normal across study groups. In 19 out of 75 ARDS patients exhibiting ACP, Vieillard-Baron *et al*. [1] previously reported a significantly reduced indexed LVEDV resulting in a decreased LV stroke index, in the presence of a normal LVEF. Due to the consistent use of a protective ventilation in our patients with moderate-to-severe ARDS, median driving pressure and median PaCO_2_ level were not significantly different between groups. The significant correlation between PaCO_2_ and sPAP levels is consistent with the effects of hypercapnia on the pulmonary vascular bed [20]. The pulmonary vasoconstriction induced by elevated PaCO_2_ level may precipitate RV failure in patients with elevated pulmonary vascular resistance, as in ARDS [21]. We previously showed that the PaCO_2_ level was the only independent factor associated with ACP, when considered as either a continuous or a binary variable [3]. Although increased LV eccentricity index at end-systole was consistent with RV afterloading [11] in our patients with ACP, irrespective of associated PFO, sPAP was not significantly higher when compared to the remaining patients. This contrasts with a greater pulmonary hypertension previously reported in ACP patients assessed using TEE [1,4]. Although we did not measure RV outflow, median RVFAC was low in our ACP patients, whether PFO was associated or not, and significantly decreased when compared to other groups, suggesting altered RV systolic function. This may have contributed to the absence of significant increase in sPAP in patients with ACP. Although median TAPSE was significantly lower in ACP patients, its reduction was less pronounced than that observed for median RVFAC. This may be related to the previously reported dependence of TAPSE to LV systolic function in ICU patients, both at baseline and following abrupt changes in loading conditions and inotropic state [22]. Noticeably, in our ACP patients who were frequently under vasopressor support, echocardiographically depicted hemodynamic changes were not associated with relevant variations of mean arterial blood pressure or lactate level. A more aggressive management of these patients who were identified early by systematic TEE assessment may explain this result [3], as reflected by the more frequent use of prone positioning, which has been shown to decrease RV afterloading [23].

Pulmonary hypertension contributes to the presence of PFO shunting in patients sustaining massive pulmonary embolism [24]. In contrast with a previous report [2], only 4% of our ARDS patients had both a PFO and ACP. Despite substantial levels of sPAP, patients with isolated PFO had a preserved RV systolic function, as reflected by median values of RVFAC and TAPSE, which were close to those measured in patients without ACP and PFO. This suggests that interatrial shunting could have a protective effect in unloading the RV submitted to abruptly increased afterload. In contrast, this potential mechanism was not operant in patients with both ACP and PFO who exhibited reduced median values of RVFAC and TAPSE, similar to those measured in patients with isolated ACP. This is presumably related to the greater severity of RV afterloading in these specific groups, which is reflected by a markedly increased LV end-systolic eccentricity index, and to the predominantly small and intermittent PFO shunting observed in our ARDS patients [3].

This multicenter study suffers from several limitations. Although this exploratory multicenter study included a large number of ARDS patients, the sample size of certain study groups is small. We did not measure RV outflow to indirectly assess pulmonary vascular resistance in our ARDS patients [25]. Accordingly, we could not substantiate the mechanism of ACP-associated RV failure, which mainly relies on acute afterloading [17]. We used LVEF as an index of LV systolic function even though this parameter is known to be load-dependent and therefore fails to reflect intrinsic myocardial contractility [26]. We did not perform serial echocardiographic assessment in all our patients to determine the course of ARDS-associated ACP and PFO. Finally, our cohort remains too small to assess the potential impact of the severity of ARDS-associated hypoxemia and hypercapnia on central hemodynamics and to confirm the deleterious effect of marked RV dilatation (that is, RVEDA/LVEDA >1) on cardiac performance, as previously suggested [27].

## Conclusions

In patients under protective mechanical ventilation with moderate-to-severe ARDS, ACP was associated with LV restriction and RV failure, whether PFO was concomitantly present or not, while LV systolic function was preserved. sPAP was significantly correlated with PaCO_2_ level. Despite a substantially increased sPAP, patients with isolated PFO shunting exhibited normal RV function. The influence of the severity of ARDS-associated hypoxemia and hypercapnia on central hemodynamics and the course of ACP and PFO in ARDS patients under protective ventilation remain to be determined in further large-scale studies.

## Key messages

- In patients under protective mechanical ventilation for moderate-to-severe acute respiratory distress syndrome, acute cor pulmonale was associated with a restricted left ventricle and impaired right ventricular systolic function, irrespective of associated patent foramen ovale
- In these patients, left ventricular stroke volume tended to be lower while left ventricular systolic function was preserved
- Despite elevated systolic pulmonary artery pressure, patients with isolated patent foramen ovale shunting had normal right ventricular systolic function.

## Abbreviations

ACP: acute cor pulmonale
ARDS: acute respiratory distress syndrome
CI: confidence interval
CVP: central venous pressure
FiO_2_: fraction of inspired oxygen
ICU: intensive care unit
LV: left ventricle
LVEDA: left ventricular end-diastolic area
LVEDV: left ventricular end-diastolic volume
LVEF: left ventricular ejection fraction
LVOT: left ventricular outflow tract
LVSV: left ventricular stroke volume
PaCO_2_: partial pressure of arterial carbon dioxide
PaO_2_: partial pressure of arterial oxygen
PEEP: positive end-expiratory pressure
PFO: patent foramen ovale
RV: right ventricle
RVEDA: right ventricular end-diastolic area
RVESA: right ventricular end-systolic area
RVFAC: right ventricular fractional area change
SD: standard deviation
sPAP: systolic pulmonary artery pressure
TAPSE: tricuspid annular plane systolic elevation
TEE: transesophageal echocardiography
TTE: transthoracic echocardiography
Vmax TR: maximal velocity of tricuspid regurgitation
V_T_: tidal volume
